# (η^4^-Cyclo­octa­tetra­ene)(η^8^-cyclo­octa­tetra­ene)iodido­tantalum(V)

**DOI:** 10.1107/S1600536814012379

**Published:** 2014-06-07

**Authors:** Pratik Verma, Victor J. Sussman, William W. Brennessel, John E. Ellis

**Affiliations:** a Department of Chemistry, 207 Pleasant Street SE, University of Minnesota, Minneapolis, MN 55455, USA

## Abstract

The title complex, [Ta(η^4^-C_8_H_8_)(η^8^-C_8_H_8_)I], lies across a crystallographic mirror plane that includes the Ta^V^ atom and the iodide ligand. One cyclo­octa­tetra­ene (cot) ring is η^4^-coordinating and is bis­ected by the mirror plane. The fold angle between the plane of the coordinating butadiene portion and the middle plane of the ring is 27.4 (4)°. An additional minor fold angle of 9.3 (7)° exists between the final plane in the ring and the middle plane. The other cot ring is η^8^-coordinating and is also cut by the mirror plane. In this case, the ring is disordered over the mirror plane, and one position is modeled with appropriate restraints and constraints with respect to distances, angles and displacement parameters (the second position is generated by symmetry). This ring is nearly planar, with an r.m.s. deviation of only 0.05 Å when all eight C atoms are included in the calculation. Pairs of inter­molecular η^8^-cot rings are parallel stacked and slightly off center, with a centroid–centroid distance of 3.652 Å. No other significant inter­molecular inter­actions are observed. The compound is of inter­est as the first structurally characterized mixed halogen–cot complex of the group 5 metals and contains the longest terminal Ta—I distance [3.0107 (5) Å] reported to date.

## Related literature   

For synthesis of the precursor tris­(naphthalene)­tantalate, see: Brennessel *et al.* (2002 ▶). For related *MX*(cot)_2_, *M* = Nb, Ta, *X* = Cl, Me, Ph, see: Schrock *et al.* (1976 ▶). For the only other structurally characterized η^8^-coordinated cyclo­octa­tetra­ene­tantalum species to date, (η-1,4-bis­(tri­methyl­sil­yl)cot)Me_3_Ta, see: Clegg & McCamley (2005 ▶). For the compound containing the previous longest terminal Ta—I distance, see: Berneri *et al.* (1998 ▶). For Zr(cot)_2_, which also contains both η^4^-cot and η^8^-cot units, see: Cloke *et al.* (1994 ▶). For a description of the Cambridge Structural Database, see: Allen (2002 ▶).
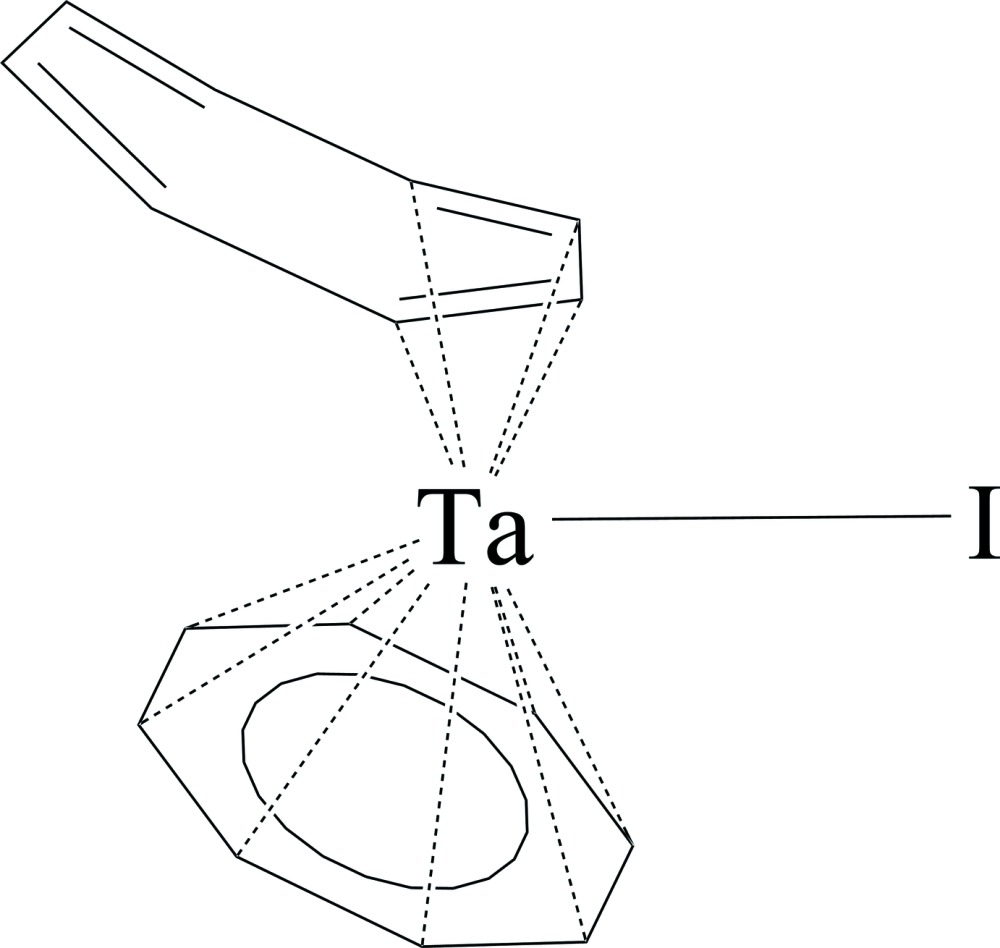


## Experimental   

### 

#### Crystal data  

[Ta(C_8_H_8_)_2_I]
*M* *_r_* = 516.14
Monoclinic, 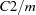

*a* = 14.3626 (14) Å
*b* = 11.0200 (11) Å
*c* = 9.3467 (9) Å
β = 113.522 (2)°
*V* = 1356.4 (2) Å^3^
*Z* = 4
Mo *K*α radiation
μ = 10.36 mm^−1^
*T* = 173 K
0.10 × 0.10 × 0.10 mm

#### Data collection  

Siemens SMART CCD platform diffractometer
Absorption correction: multi-scan (*SADABS*; Sheldrick, 2012 ▶) *T*_min_ = 0.318, *T*_max_ = 0.431
8110 measured reflections
1636 independent reflections
1520 reflections with *I* > 2σ(*I*)
*R*_int_ = 0.020

#### Refinement  

*R*[*F* ^2^ > 2σ(*F* ^2^)] = 0.017
*wR*(*F* ^2^) = 0.040
*S* = 1.08
1636 reflections
97 parameters
96 restraints
H-atom parameters constrained
Δρ_max_ = 1.04 e Å^−3^
Δρ_min_ = −0.72 e Å^−3^

### 

Data collection: *SMART* (Bruker, 2003 ▶); cell refinement: *SAINT* (Bruker, 2003 ▶); data reduction: *SAINT*; program(s) used to solve structure: *SHELXS97* (Sheldrick, 2008 ▶); program(s) used to refine structure: *SHELXL2014* (Sheldrick, 2008 ▶); molecular graphics: *SHELXTL* (Sheldrick, 2008 ▶); software used to prepare material for publication: *SHELXTL*.

## Supplementary Material

Crystal structure: contains datablock(s) I, global. DOI: 10.1107/S1600536814012379/lh5707sup1.cif

Structure factors: contains datablock(s) I. DOI: 10.1107/S1600536814012379/lh5707Isup2.hkl

CCDC reference: 1005544

Additional supporting information:  crystallographic information; 3D view; checkCIF report

## Figures and Tables

**Fig. 1.**
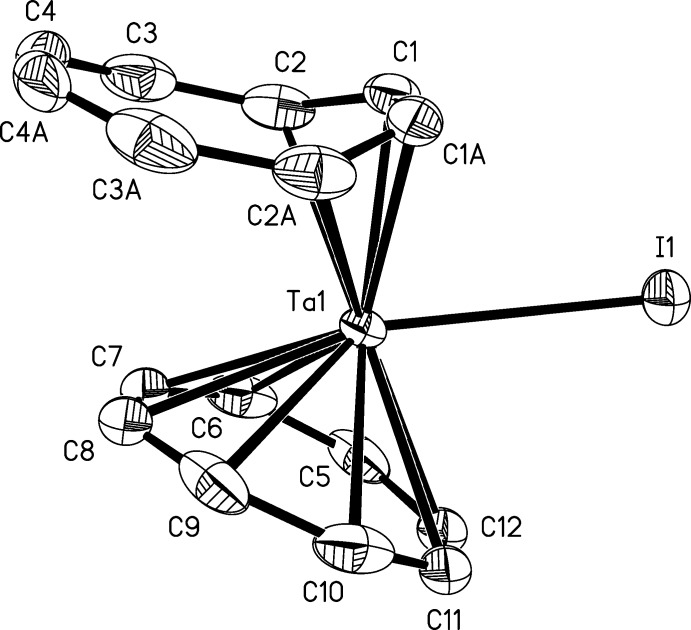
The structure of the molecule without hydrogen atoms, showing the atom numbering. Only one of the two positions of the disordered ligand is shown. Displacement ellipsoids are drawn at the 50% probability level.

## supplementary crystallographic information

### S1. Comment

Bis(cyclooctatetraene)iodotantalum, TaI(cot)_2_, is the first structurally authenticated mixed halogen-cot complex of a group 5 metal (Fig. 1). In prior work, reaction of MCl_5_, *M* = Nb, Ta, with two equivalents of K_2_[cot] was suggested to afford the chloro analogs, MCl(cot)_2_ (Schrock *et al.*, 1976). Although these compounds were not isolated or fully characterized in solution, the ^1^H NMR spectrum of the niobium complex in CD_2_Cl_2_ at 273 K showed two singlets in a 1:1 ratio at δ 5.49 and 6.24 p.p.m., in excellent agreement with those observed for TaI(cot)_2_ in THF-*d*_8_, see below. Thus, our study provides additional support for the existence of NbCl(cot)_2_ and TaCl(cot)_2_ and suggests that the latter contain η^4^– and η^8^-cot groups, as observed in TaI(cot)_2_. Structural features of the η^4^-cot group in TaI(cot)_2_ are nearly identical to the one present in [Ta(cot)_3_]^1-^ (Brennessel *et al.*, 2002). For example, the η^4^-cot-(centroid)-Ta distances in these two species are 2.023 and 2.025 Å, respectively. The only other structurally characterized η^8^-cot-Nb or –Ta complex for comparison is TaMe_3_(cot") (Clegg & McCamley, 2005), where cot" = 1,4-bis(trimethylsilyl)cyclooctatetraene, in which the η^8^-cot"-(centroid)-Ta distance, 1.627 Å, is slightly longer than the corresponding distance in TaI(cot)_2_, 1.606 Å, likely owing to the more bulky nature of cot" *versus* cot. The only surprising feature in the structure of TaI(cot)_2_ is the unusually long Ta–I distance of 3.0108 (5) Å, which exceeds the prior "record" value of 2.9621 (7) Å, observed in the 7-coordinate complex, TaI(CO)_4_(tmen), tmen = 1,2-bis(dimethylamino)ethane (Berneri *et al.*, 1998). Because the η^4^ and η^8^ units in the formally Zr(IV) complex, Zr(cot)_2_ (Cloke *et al.*, 1994) and TaI(cot)_2_ are quite analogous in structure and binding to the respective metals, the tantalum complex is best formulated to contain Ta(V).

### S2. Experimental

Under argon, a yellow-orange solution of [Na(THF)_6_][Ta(η^4^-C_10_H_8_)_3_] (Brennessel *et al.*, 2002) in tetrahydrofuran (THF) was combined with three equiv. of 1,3,5,7-cyclooctatetraene (cot) at room temperature and stirred for 24 h to provide a deep purple solution of [Na(THF)*_x_*][Ta(cot)_3_]. Cation exchange of the latter (Brennessel *et al.*, 2002) with bis(triphenylphosphine)iminium chloride, PPN^+^Cl^-^, in ethanol under argon, provided a purple-brown precipitate of [PPN][Ta(cot)_3_] in 67% yield. The latter was reacted with one equiv. of elemental iodine in THF at 205 K, followed by warming to room temperature over a four hour period. Following filtration to remove poorly soluble PPN^+^I^-^, a deep red-purple solid of composition TaI(cot)_2_ was isolated in 49% yield. ^1^H NMR (300 MHz, 298 K, THF-*d*_8_, δ, p.p.m.) 5.53 (s, 8H, cot), 6.27 (s, 8H, cot). Suitable single crystals of the product were grown from a pentane-layered THF solution over a period of several days at 273 K.

### S3. Refinement

The η^8^-cot ligand is modeled as disordered over a crystallographic mirror plane (50:50). Bond lengths and angles in the η^8^-cot ligand were restrained to be similar to those of their neighbors. Anisotropic displacement parameters for pairs of atoms opposite to each other were constrained to be equivalent, and those of symmetry-related atom pair C11 and C12 were heavily restrained to be similar. H atoms were placed geometrically and treated as riding atoms: C—H = 0.95 Å with *U*_iso(_H) = 1.2*U*_eq_(C).

### Crystal data

**Table:**

[Ta(C_8_H_8_)_2_I]	*F*(000) = 952
*M**_r_* = 516.14	*D*_x_ = 2.527 Mg m^−^^3^
Monoclinic, *C*2/*m*	Mo *K*α radiation, λ = 0.71073 Å
*a* = 14.3626 (14) Å	Cell parameters from 937 reflections
*b* = 11.0200 (11) Å	θ = 2.4–27.5°
*c* = 9.3467 (9) Å	µ = 10.36 mm^−^^1^
β = 113.522 (2)°	*T* = 173 K
*V* = 1356.4 (2) Å^3^	Block, red-purple
*Z* = 4	0.10 × 0.10 × 0.10 mm

### Data collection

**Table:**

Siemens SMART CCD platform diffractometer	1520 reflections with *I* > 2σ(*I*)
Radiation source: normal-focus sealed tube	*R*_int_ = 0.020
ω scans	θ_max_ = 27.5°, θ_min_ = 2.4°
Absorption correction: multi-scan (*SADABS*; Sheldrick, 2012)	*h* = −18→18
*T*_min_ = 0.318, *T*_max_ = 0.431	*k* = −14→14
8110 measured reflections	*l* = −12→12
1636 independent reflections	

### Refinement

**Table:**

Refinement on *F*^2^	Primary atom site location: structure-invariant direct methods
Least-squares matrix: full	Secondary atom site location: difference Fourier map
*R*[*F*^2^ > 2σ(*F*^2^)] = 0.017	Hydrogen site location: inferred from neighbouring sites
*wR*(*F*^2^) = 0.040	H-atom parameters constrained
*S* = 1.08	*w* = 1/[σ^2^(*F*_o_^2^) + (0.018*P*)^2^ + 5.3173*P*] where *P* = (*F*_o_^2^ + 2*F*_c_^2^)/3
1636 reflections	(Δ/σ)_max_ = 0.003
97 parameters	Δρ_max_ = 1.04 e Å^−^^3^
96 restraints	Δρ_min_ = −0.72 e Å^−^^3^

### Special details

**Table:**

Geometry. All e.s.d.'s (except the e.s.d. in the dihedral angle between two l.s. planes) are estimated using the full covariance matrix. The cell e.s.d.'s are taken into account individually in the estimation of e.s.d.'s in distances, angles and torsion angles; correlations between e.s.d.'s in cell parameters are only used when they are defined by crystal symmetry. An approximate (isotropic) treatment of cell e.s.d.'s is used for estimating e.s.d.'s involving l.s. planes.
Refinement. The η^8^-cot ligand is modeled as disordered over a crystallographic mirror plane (50:50). Bond lengths and angles in the η^8^-cot ligand were restrained to be similar to those of their neighbors. Anisotropic displacement parameters for pairs of atoms opposite to each other were constrained to be equivalent, and those of symmetry-related atom pair C11 and C12 were heavily restrained to be similar.H atoms were placed geometrically and treated as riding atoms: C—H = 0.95 Å with *U*_iso(_H) = 1.2*U*_eq_(C).

### Fractional atomic coordinates and isotropic or equivalent isotropic displacement parameters (Å^2^)

**Table:**

	*x*	*y*	*z*	*U*_iso_*/*U*_eq_	Occ. (<1)
Ta1	0.28337 (2)	0.5000	0.16766 (2)	0.01915 (6)	
I1	0.26793 (2)	0.5000	−0.16320 (4)	0.02953 (8)	
C1	0.1203 (2)	0.4365 (4)	0.0278 (4)	0.0312 (8)	
H1	0.1043	0.4003	−0.0715	0.037*	
C2	0.1398 (3)	0.3549 (3)	0.1493 (5)	0.0356 (9)	
H2	0.1770	0.2859	0.1413	0.043*	
C3	0.1160 (3)	0.3522 (4)	0.2844 (5)	0.0455 (11)	
H3	0.1214	0.2729	0.3271	0.055*	
C4	0.0875 (3)	0.4358 (5)	0.3660 (5)	0.0531 (13)	
H4	0.0626	0.4012	0.4371	0.064*	
C5	0.4245 (10)	0.3628 (8)	0.2362 (15)	0.039 (3)	0.5
H5	0.4396	0.2896	0.1962	0.047*	0.5
C6	0.3753 (8)	0.3428 (9)	0.3361 (12)	0.038 (4)	0.5
H6	0.3574	0.2602	0.3398	0.046*	0.5
C7	0.3473 (7)	0.4163 (9)	0.4297 (10)	0.042 (2)	0.5
H7	0.3298	0.3703	0.5013	0.050*	0.5
C8	0.3375 (6)	0.5382 (8)	0.4486 (9)	0.042 (3)	0.5
H8	0.3127	0.5560	0.5266	0.051*	0.5
C9	0.3558 (12)	0.6407 (8)	0.3790 (17)	0.039 (3)	0.5
H9	0.3318	0.7123	0.4097	0.047*	0.5
C10	0.4007 (7)	0.6649 (8)	0.2747 (12)	0.038 (4)	0.5
H10	0.4011	0.7488	0.2518	0.046*	0.5
C11	0.4453 (7)	0.5925 (8)	0.1965 (11)	0.042 (2)	0.5
H11	0.4749	0.6393	0.1400	0.050*	0.5
C12	0.4570 (5)	0.4667 (6)	0.1811 (8)	0.042 (3)	0.5
H12	0.4949	0.4481	0.1208	0.051*	0.5

### Atomic displacement parameters (Å^2^)

**Table:**

	*U*^11^	*U*^22^	*U*^33^	*U*^12^	*U*^13^	*U*^23^
Ta1	0.01532 (9)	0.02046 (10)	0.01810 (10)	0.000	0.00291 (7)	0.000
I1	0.03124 (17)	0.03438 (18)	0.02409 (16)	0.000	0.01224 (13)	0.000
C1	0.0182 (15)	0.045 (2)	0.0268 (17)	−0.0062 (15)	0.0053 (13)	−0.0106 (16)
C2	0.0215 (17)	0.0272 (18)	0.049 (2)	−0.0006 (14)	0.0037 (16)	−0.0047 (16)
C3	0.0277 (19)	0.047 (2)	0.047 (2)	−0.0136 (18)	−0.0006 (18)	0.020 (2)
C4	0.0250 (18)	0.105 (4)	0.027 (2)	−0.011 (2)	0.0080 (16)	0.017 (2)
C5	0.024 (6)	0.055 (6)	0.029 (6)	0.012 (5)	0.001 (3)	−0.019 (5)
C6	0.027 (9)	0.027 (3)	0.045 (13)	−0.007 (3)	−0.002 (5)	0.008 (3)
C7	0.0205 (16)	0.079 (8)	0.0247 (18)	−0.008 (2)	0.0078 (14)	0.001 (2)
C8	0.0210 (15)	0.079 (8)	0.0248 (17)	−0.008 (2)	0.0071 (13)	0.000 (2)
C9	0.024 (6)	0.055 (6)	0.029 (6)	0.012 (5)	0.001 (3)	−0.019 (5)
C10	0.027 (9)	0.027 (3)	0.045 (13)	−0.007 (3)	−0.002 (5)	0.008 (3)
C11	0.0205 (16)	0.079 (8)	0.0247 (18)	−0.008 (2)	0.0078 (14)	0.001 (2)
C12	0.0210 (15)	0.079 (8)	0.0248 (17)	−0.008 (2)	0.0071 (13)	0.000 (2)

### Geometric parameters (Å, º)

**Table:**

Ta1—C1	2.290 (3)	C3—H3	0.9500
Ta1—C1^i^	2.290 (3)	C4—C4^i^	1.416 (11)
Ta1—C6	2.359 (10)	C4—H4	0.9500
Ta1—C9	2.397 (13)	C5—C6	1.396 (9)
Ta1—C5	2.402 (12)	C5—C12	1.410 (9)
Ta1—C10	2.408 (9)	C5—H5	0.9500
Ta1—C7	2.429 (9)	C6—C7	1.365 (9)
Ta1—C11	2.454 (9)	C6—H6	0.9500
Ta1—C8	2.459 (8)	C7—C8	1.369 (9)
Ta1—C12	2.474 (7)	C7—H7	0.9500
Ta1—C2	2.560 (4)	C8—C9	1.381 (9)
Ta1—C2^i^	2.560 (4)	C8—H8	0.9500
Ta1—I1	3.0107 (5)	C9—C10	1.392 (9)
C1—C2	1.386 (5)	C9—H9	0.9500
C1—C1^i^	1.400 (8)	C10—C11	1.399 (9)
C1—H1	0.9500	C10—H10	0.9500
C2—C3	1.435 (6)	C11—C12	1.410 (9)
C2—H2	0.9500	C11—H11	0.9500
C3—C4	1.359 (7)	C12—H12	0.9500
			
C1—Ta1—C1^i^	35.6 (2)	C7—Ta1—I1	151.54 (19)
C1—Ta1—C6	109.2 (3)	C11—Ta1—I1	77.9 (2)
C1^i^—Ta1—C6	141.1 (2)	C8—Ta1—I1	163.56 (16)
C1—Ta1—C9	132.6 (3)	C12—Ta1—I1	73.25 (16)
C1^i^—Ta1—C9	106.3 (3)	C2—Ta1—I1	101.77 (9)
C6—Ta1—C9	89.0 (3)	C2^i^—Ta1—I1	101.77 (9)
C1—Ta1—C5	121.5 (3)	C2—C1—C1^i^	130.4 (2)
C1^i^—Ta1—C5	155.1 (3)	C2—C1—Ta1	84.5 (2)
C6—Ta1—C5	34.1 (2)	C1^i^—C1—Ta1	72.20 (10)
C9—Ta1—C5	98.3 (3)	C2—C1—H1	114.8
C1—Ta1—C10	148.7 (2)	C1^i^—C1—H1	114.8
C1^i^—Ta1—C10	113.2 (2)	Ta1—C1—H1	118.6
C6—Ta1—C10	99.5 (3)	C1—C2—C3	133.9 (4)
C9—Ta1—C10	33.7 (2)	C1—C2—Ta1	62.89 (19)
C5—Ta1—C10	89.3 (3)	C3—C2—Ta1	115.0 (2)
C1—Ta1—C7	110.4 (3)	C1—C2—H2	113.0
C1^i^—Ta1—C7	125.5 (2)	C3—C2—H2	113.0
C6—Ta1—C7	33.1 (2)	Ta1—C2—H2	92.4
C9—Ta1—C7	63.2 (3)	C4—C3—C2	135.3 (4)
C5—Ta1—C7	63.4 (3)	C4—C3—H3	112.4
C10—Ta1—C7	87.0 (3)	C2—C3—H3	112.4
C1—Ta1—C11	154.0 (2)	C3—C4—C4^i^	132.7 (3)
C1^i^—Ta1—C11	130.1 (2)	C3—C4—H4	113.7
C6—Ta1—C11	88.8 (3)	C4^i^—C4—H4	113.7
C9—Ta1—C11	63.9 (3)	C6—C5—C12	134.7 (8)
C5—Ta1—C11	64.5 (3)	C6—C5—Ta1	71.3 (5)
C10—Ta1—C11	33.4 (2)	C12—C5—Ta1	76.0 (6)
C7—Ta1—C11	95.0 (3)	C6—C5—H5	112.7
C1—Ta1—C8	118.50 (19)	C12—C5—H5	112.7
C1^i^—Ta1—C8	111.9 (2)	Ta1—C5—H5	136.9
C6—Ta1—C8	63.8 (3)	C7—C6—C5	133.8 (8)
C9—Ta1—C8	33.0 (2)	C7—C6—Ta1	76.2 (6)
C5—Ta1—C8	87.1 (3)	C5—C6—Ta1	74.6 (6)
C10—Ta1—C8	63.7 (3)	C7—C6—H6	113.1
C7—Ta1—C8	32.5 (2)	C5—C6—H6	113.1
C11—Ta1—C8	86.0 (3)	Ta1—C6—H6	129.8
C1—Ta1—C12	139.89 (19)	C6—C7—C8	137.5 (9)
C1^i^—Ta1—C12	148.81 (18)	C6—C7—Ta1	70.7 (6)
C6—Ta1—C12	64.7 (2)	C8—C7—Ta1	75.0 (6)
C9—Ta1—C12	87.5 (4)	C6—C7—H7	111.3
C5—Ta1—C12	33.6 (2)	C8—C7—H7	111.3
C10—Ta1—C12	64.3 (3)	Ta1—C7—H7	144.5
C7—Ta1—C12	85.7 (3)	C7—C8—C9	133.9 (9)
C11—Ta1—C12	33.2 (2)	C7—C8—Ta1	72.5 (6)
C8—Ta1—C12	95.1 (2)	C9—C8—Ta1	71.0 (6)
C1—Ta1—C2	32.60 (13)	C7—C8—H8	113.1
C1^i^—Ta1—C2	62.58 (13)	C9—C8—H8	113.1
C6—Ta1—C2	78.9 (2)	Ta1—C8—H8	143.0
C9—Ta1—C2	122.1 (2)	C8—C9—C10	135.7 (8)
C5—Ta1—C2	101.3 (2)	C8—C9—Ta1	76.0 (6)
C10—Ta1—C2	155.6 (2)	C10—C9—Ta1	73.6 (6)
C7—Ta1—C2	78.5 (3)	C8—C9—H9	112.2
C11—Ta1—C2	165.8 (2)	C10—C9—H9	112.2
C8—Ta1—C2	94.66 (19)	Ta1—C9—H9	134.1
C12—Ta1—C2	132.81 (18)	C9—C10—C11	133.9 (9)
C1—Ta1—C2^i^	62.58 (13)	C9—C10—Ta1	72.7 (6)
C1^i^—Ta1—C2^i^	32.60 (13)	C11—C10—Ta1	75.1 (6)
C6—Ta1—C2^i^	136.4 (2)	C9—C10—H10	113.0
C9—Ta1—C2^i^	74.0 (3)	C11—C10—H10	113.0
C5—Ta1—C2^i^	169.2 (3)	Ta1—C10—H10	135.0
C10—Ta1—C2^i^	88.2 (2)	C10—C11—C12	135.4 (8)
C7—Ta1—C2^i^	106.0 (2)	C10—C11—Ta1	71.5 (5)
C11—Ta1—C2^i^	116.8 (2)	C12—C11—Ta1	74.2 (5)
C8—Ta1—C2^i^	82.4 (2)	C10—C11—H11	112.3
C12—Ta1—C2^i^	149.84 (18)	C12—C11—H11	112.3
C2—Ta1—C2^i^	77.28 (17)	Ta1—C11—H11	141.1
C1—Ta1—I1	76.97 (9)	C11—C12—C5	133.7 (8)
C1^i^—Ta1—I1	76.97 (9)	C11—C12—Ta1	72.6 (5)
C6—Ta1—I1	118.5 (2)	C5—C12—Ta1	70.4 (6)
C9—Ta1—I1	132.5 (2)	C11—C12—H12	113.1
C5—Ta1—I1	89.0 (3)	C5—C12—H12	113.1
C10—Ta1—I1	100.3 (2)	Ta1—C12—H12	143.8
			
C1^i^—C1—C2—C3	38.6 (5)	C7—C8—C9—C10	−8 (3)
Ta1—C1—C2—C3	99.6 (4)	Ta1—C8—C9—C10	−47.7 (16)
C1^i^—C1—C2—Ta1	−60.97 (17)	C7—C8—C9—Ta1	39.5 (14)
C1—C2—C3—C4	−18.5 (8)	C8—C9—C10—C11	1 (2)
Ta1—C2—C3—C4	57.1 (5)	Ta1—C9—C10—C11	−47.7 (12)
C2—C3—C4—C4^i^	−13.5 (6)	C8—C9—C10—Ta1	48.4 (16)
C12—C5—C6—C7	−7 (2)	C9—C10—C11—C12	6 (2)
Ta1—C5—C6—C7	−52.8 (12)	Ta1—C10—C11—C12	−41.4 (14)
C12—C5—C6—Ta1	46.1 (13)	C9—C10—C11—Ta1	47.0 (12)
C5—C6—C7—C8	14 (2)	C10—C11—C12—C5	3 (2)
Ta1—C6—C7—C8	−37.9 (15)	Ta1—C11—C12—C5	−38.2 (13)
C5—C6—C7—Ta1	52.2 (12)	C10—C11—C12—Ta1	40.7 (14)
C6—C7—C8—C9	−2 (3)	C6—C5—C12—C11	−6 (2)
Ta1—C7—C8—C9	−39.1 (15)	Ta1—C5—C12—C11	38.8 (13)
C6—C7—C8—Ta1	36.9 (15)	C6—C5—C12—Ta1	−44.7 (13)

Symmetry code: (i) *x*, −*y*+1, *z*.
